# Rapid In Vivo Screening of Monoclonal Antibody Cocktails Using Hydrodynamic Delivery of DNA-Encoded Modified Antibodies

**DOI:** 10.3390/biomedicines13030637

**Published:** 2025-03-05

**Authors:** Hugues Fausther-Bovendo, George (Giorgi) Babuadze, Teodora Ivanciuc, Birte Kalveram, Yue Qu, Jihae Choi, Allison McGeer, Mario Ostrowski, Samira Mubareka, Ami Patel, Roberto P. Garofalo, Robert Kozak, Gary P. Kobinger

**Affiliations:** 1 Department of Microbiology and Immunology, University of Texas Medical Branch, Galveston, TX 77555, USAgakobing@utmb.edu (G.P.K.); 2 The Sealy Institute of Drug Discovery, University of Texas Medical Branch, Galveston, TX 77555, USA; 3 Sealy Center on Lung Disease, Inflammation and Remodeling, University of Texas Medical Branch, Galveston, TX 77555, USA; 4 The Wistar Institute, Philadelphia, PA 19104, USA; 5 Department of Laboratory Medicine and Pathobiology, University of Toronto, Toronto, ON M5S 3K3, Canadarob.kozak@sunnybrook.ca (R.K.); 6 Department of Microbiology, Sinai Health System, University Health Network, Toronto, ON M5G 2C4, Canada; 7 Department of Medicine, University of Toronto, Toronto, ON M5S 1A1, Canada; 8 Department of Laboratory Medicine and Molecular Diagnostics, Division of Microbiology, Sunnybrook Health Sciences Centre, Toronto, ON M4N 3M5, Canada; 9 Biological Sciences Platform, Sunnybrook Research Institute at Sunnybrook Health Sciences Centre, University of Toronto, Toronto, ON M4N 3M5, Canada; 10 Galveston National Laboratory, University of Texas Medical Branch, Galveston, TX 77555, USA

**Keywords:** DNA-encoded antibody, monoclonal antibodies cocktail, in vivo screening

## Abstract

**Background:** Monoclonal antibodies (mAbs) are potent treatment options for infectious diseases. The rapid isolation and in vivo validation of therapeutic mAb candidates, including mAb cocktails, are essential to combat novel or rapidly mutating pathogens. The rapid selection and production of mAb candidates in sufficient amount and quality for preclinical studies are a major limiting step in the mAb development pipeline. **Methods:** Here, we developed a method to facilitate the screening of therapeutic mAbs in mouse models. Four conventional mAbs were transformed into single-chain variable fragments fused to the fragment crystallizable (Fc) region of a human IgG1 (scFv-IgG). These scFv-IgG were expressed individually or as a cocktail in vitro and in mice following transfection or hydrodynamic delivery of the corresponding plasmids. **Results:** This method induced high expression of all scFv-IgG and provided protection in two murine infection models. **Conclusions:** This study highlights the benefits of this approach for the rapid, low-cost screening of therapeutic mAb candidates.

## 1. Introduction

Therapeutic monoclonal antibodies (mAbs) have demonstrated significant potency against infectious diseases in numerous clinical and pre-clinical studies. Notably, Palivizumab and Nirsevimab are approved for prophylactic use against respiratory syncytial virus (RSV) in infants [1,2,3,4]. Various mAbs received emergency use approval from the US Food and Drug Administration (FDA) for the treatment of coronavirus disease 2019 (COVID-19) patients with mild and moderate symptoms [5,6]. Additionally, in a clinical trial, mAb114 and the REGN-EB3 cocktail improved survival rates in Ebola virus-infected individuals [7]. Despite their efficacy, the impact of therapeutic mAbs on emerging pathogens remains limited. The rapid spread of novel or reemerging pathogens, such as during the COVID-19 pandemic, the 2022 mpox outbreak, and the 2014 Ebola virus outbreak, underscores the need for expedited mAb candidates’ isolation and screening for therapeutic mAbs to significantly impact the fight against emerging pathogens [8,9]. Furthermore, escape variants resistant to existing countermeasures can also arise, especially during prolonged outbreaks. The binding affinity of first-generation therapeutic antibodies targeting the severe acute respiratory syndrome coronavirus 2 (SARS-CoV-2) spike (S) protein rapidly declined as waves of variants of concern arose, leading to the loss of their emergency use authorization [10,11].

While numerous platforms can rapidly isolate human mAbs, no in vitro assay can predict their in vivo efficacy, and preclinical studies are required following in vitro screening. Depending on the animal model, between 100 µg and 1 mg of antibody per dose per mouse or between 5 and 12 mg of antibody per dose per guinea pig is required to assess the therapeutic efficacy of mAb candidates [12,13,14,15,16]. Subsequent experiments are often required for the development of therapeutic mAb cocktails [14,17,18]. The production of the necessary amount of each of the mAb candidates is a rate-limiting step. In contrast, free plasmid DNA can be readily produced without any specialized equipment using commercially available kits. Unlike mAbs, plasmid DNA does not irreversibly aggregate at high concentrations and is highly stable at room temperature [19,20]. The intramuscular, intraperitoneal or sub-cutaneous administration of naked plasmid DNA leads to low transgene expression levels. Combinations of various formulations such as hyaluronidase and Pluronic block copolymers as well as delivery devices including gene guns, needle free delivery systems, and electroporators can significantly increase in vivo transgene expression following DNA injection [21,22,23]. However, these DNA delivery systems are not commercially available. In contrast, high transgene expression was demonstrated following hydrodynamic delivery (HD) of naked plasmid DNA via the tail vein or the retroorbital route. HD does not require any specific equipment and only requires a limited amount (10 µg or less) of plasmid DNA to induce robust transgene expression [24,25]. Finally, it is worth noting that the co-injection of multiple DNA-encoded antibodies (DMAb) generates a high proportion of non-functional mAbs with improper heavy and light chain pairing. As a result, multiple injections, one per DMAb, are required to screen mAb cocktails. However, injections into multiple sites can be challenging in smaller rodents, especially when electroporation is necessary.

Here, we described a platform for the rapid screening of both individual mAb candidates and mAb cocktails in mouse models. As a proof of concept, four mAbs, targeting the respiratory syncytial virus (RSV), the human metapneumovirus (HMPV), the severe acute respiratory syndrome virus (SARS) and/or SARS-CoV-2, were modified into single-chain variable fragments fused to the fragment crystallizable (Fc) region of a human IgG1 (scFv-IgG). Co-expression of these four scFv-IgG was demonstrated following the co-transfection of human embryonic kidney (HEK) 293 cells with a cocktail of four plasmids encoding each of the scFv-IgG (DNA-scFv-IgG). Next, circulating scFv-IgG levels were measured via Elisa following HD of individual or a cocktail of DNA-scFv-IgG. Finally, we tested the therapeutic efficacy of a cocktail of these four DNA-scFv-IgG delivered hydrodynamically in the mouse model of SARS-CoV-2 and RSV infection.

## 2. Materials and Methods

### 2.1. Human Samples

PBMCs from COVID-19 convalescent individuals were obtained during the first quarter of 2020, from Toronto (Canada). This study was conducted in accordance with the Declaration of Helsinki, and the protocol was approved by the Ethics Committee of Sunnybrook Health Sciences (Project identification number 149-1994) on 2 March 2020. Ethical approval was obtained prior to sample collection, and all participants signed individual inform consent.

### 2.2. Animal Experiments

Animal experiments were approved by the Animal Care and Use Committee at the University of Texas Medical Branch (protocol #2307043, 2401002) and were performed in accordance with the guidelines of the American Association for Laboratory Animal Science.

### 2.3. Isolation of SARS-CoV-2 Specific mAb

Plant-derived SARS-CoV-2 (hCoV-19/USA/CA2/2020) virus-like particles (VLPs) were provided by Medicago (Quebec, QC, Canada) [26]. VLPs were biotinylated using EZ-Link™ Sulfo-NHS-Biotin (ThermoFisher Scientific, Burlington, ON, Canada). PBMCs from COVID-19 convalescent individuals were thawed and stained using 0.2 µg of biotinylated VLPs. Samples were stained using Fixable Viability Dye eFluor (ThermoFisher Scientific), streptavidin Alexa 488 (Biolegend, San Jose, CA, USA), and lineage markers against CD14 (M5E2), CD3 (SP34-2), CD19 (HIB19), IgG (G18-145) and IgM (G20-127), all from BD Biosciences (San Jose, CA, USA). After washing, SARS-CoV-2-specific B cells were individually sorted using a FACSARIA Fusion (BD Biosciences). Sorted cells were cultured for 2 weeks, as previously described [27]. Both supernatant and cells from these cultures were collected for further analysis.

### 2.4. Total Human IgG-Specific ELISA

To measure antibody production, 96-well plates were coated overnight with 50 ng of unlabeled goat anti-human IgG (Mandel Scientific, Guelph, ON, Canada). After washing and blocking with PBS and 5% milk, diluted supernatants were incubated for 1 h at 37 °C. Plates were washed and incubated for 1 h with HRP-conjugated anti-human IgG (Mandel Scientific). Absorbance was measured at 405, after addition of 50 µL of ABTS substrate (Mandel Scientific). To quantify the amount of human IgG, serial dilutions of a human IgG Isotype Control (ThermoFisher Scientific) were used as a standard curve.

### 2.5. RT-PCR and Sequencing of mAb

The variable sequences of SARS-CoV-2-specific mAbs were amplified by nested RT-PCR and sequenced as previously described [28], except that total RNA was extracted from specific B cell pellets using QIAzol Lysis Reagent (Qiagen, Toronto, ON, Canada) according to the manufacturer’s instructions. PCR fragments were sequenced using the Sanger techniques by the sequencing platform at the CHU-Université Laval.

### 2.6. Production of scFv-IgG

Codon-optimized scFv fragments, in the VL-VH orientation separated by a glycine serine linker (G_4_S)_3_ [29,30] and fused to a human IgG1 fragment, were synthesized (Genescript, Piscataway, NJ, USA). These fragments were then cloned into pIDV-II, a novel plasmid [31], using standard cloning techniques. HEK293 cells (ATCC) were transfected using lipofectamine 2000 (ThermoFisher Scientific) with the generated plasmids and supernatants collected 4 days later. Plasmid DNA expressing conventional REGN10933 [18], REGN10987 [18], and C135 [32] was used as control. Supernatant levels of scFv-IgG or conventional mAbs were quantified using the total human IgG Elisa described above.

### 2.7. SARS-CoV-2, SARS, RSV and HMPV-Specific ELISA

Wells of a 96-well plate were coated overnight with 50 ng of target antigens per well. The following reagent was obtained through BEI Resources, NIAID, NIH: S protein (Stabilized) from SARS-CoV-2, Wuhan-Hu-1 (NR-53937); S protein RBD from SARS-CoV-2, Wuhan-Hu-1 (NR-53800); S protein RBD from SARS-CoV-2, Beta Variant (NR-55278); and the F Glycoprotein from RSV B, Strain 18537 (NR-58649). SARS S protein (40634-V08B) and HMPV F protein (REC31970) were purchased from Cedarlanes lab. After washing and blocking with PBS and 5% milk, wells were incubated with culture supernatant for 1 h at 37 °C. Following additional washes, wells were incubated with 15 ng per well of HRP-conjugated goat anti-human IgG (Mandel Scientific). After additional washes, wells were incubated with ABTS substrate (Mandel Scientific), and the absorbance was read at 405 nm. For quantification, standard curves made of serial dilutions of transfection supernatant adjusted to 200 ng/mL of antibodies were used.

### 2.8. SARS-CoV-2 Neutralization Assay

SARS-CoV-2 neutralization experiments were performed in the Wistar Institute BSL3 Facility. The following reagent was deposited by the Centers for Disease Control and Prevention and obtained through BEI Resources, NIAID, NIH: SARS-CoV-2, Isolate USA-WA1/2020. Vero cells (ATCC; CCL-81), maintained in DMEM supplemented with 10% FBS, were seeded at 20,000 cells/well in a 96-well plate in DMEM + 1% FBS + 1% Penn/Strep the day before the assay. Serial dilutions of antibody were incubated for 1 h at RT with 300 TCID50/mL of virus before the mixture was transferred to seeded Vero cells and incubated for 5 days. Viral titer was determined as the presence or absence of CPE and calculated using the Reed and Muench method.

### 2.9. scFv-IgG In Vivo Level

Further, 10 µg of individual DNA-scFv-IgG or 20 µg of a cocktail of the 4 DNA-scFv-IgG was administered to 5- to 6-week-old female BALB/c mice (Jackson Laboratory, Bar Harbor, ME, USA). The injection was performed retroorbitally on fully anesthetized mice in a total saline volume corresponding to 10% of the mice body weight, with each injection lasting approximately 12 s [25]. Mice were bled via the saphenous vein, 7 days before, then 1, 8, and 15 days after the injection. Animals were exsanguinated on day 23. The scFv-IgG serum levels were measured by ELISA, as described above.

### 2.10. Mice Challenge Studies

Five- to six-week-old female human ACE2 transgenic mice (K18-ACE2, strain 034860) were from Jackson Laboratory. Animals were acclimatized for at least 3 days before the start of any experiment. Mice were randomly assigned to different groups at the start of the experiment. Groups of 6 animals received either 20 µg of a control DNA-scFv-IgG or 5 µg each of the 4 described DNA-scFv-IgG, three days before challenge. HD of DNA-scFv-IgG occurred via the retroorbital route, as described above [25]. Naïve mice or mice treated intraperitoneally with 200 µg (~10 mg/kg) of sotrovimab (Ichor Bio, Wantage, UK), 24 h before challenge, were used as negative and positive control, respectively. All animals were challenged intranasally with 6 × 10^4^ TCID50 SARS-CoV-2 (USA-WA1/2020), kindly provided by Dr. Tseng, Chien-Te, in the BSL-3 animal facility at the University of Texas Medical Branch. Animals were inspected for signs of diseases and weighed daily for 21 days post-challenge. Investigators were not blinded to animal scoring.

For RSV challenge, 5- to 6-week-old female BALB/c mice were taken from Jackson Laboratory. As described above, groups of 5 animals were injected with either 20 µg control DNA-scFv-IgG, 20 µg of DNA-scFv-IgG cocktail or 100 µg (5 mg/kg) of Palivizumab (Ichor Bio). HD of DNA-scFv-IgG occurred 3 days before challenge, while Palivizumab was injected intraperitoneally 1 day before challenge. After intranasal challenge using 5 × 10^6^ PFU of RSV (strain pool G493), mice were weighed and monitored daily for signs of diseases. All animals were culled 4 days after challenge. Lung viral titers were measured using plaque assay, as previously described [33].

### 2.11. Statistical Analysis

Before performing animal experiments, the PASS 2023 software (version 23.0.2) was used by our biostatistics office to ensure that all animal experiments were sufficiently powered. Statistical analysis was performed using Graphpad prism 10. Differences in survival rates were compared using the Log-rank (Mantel-Cox) test. Lung viral titers were compared using a one-way ANOVA followed by a Dunnett’s multiple comparisons test. In this study, no data points were excluded from analysis.

## 3. Results

### 3.1. Construction of scFv-IgG from Conventional mAbs

**Figure 1:**
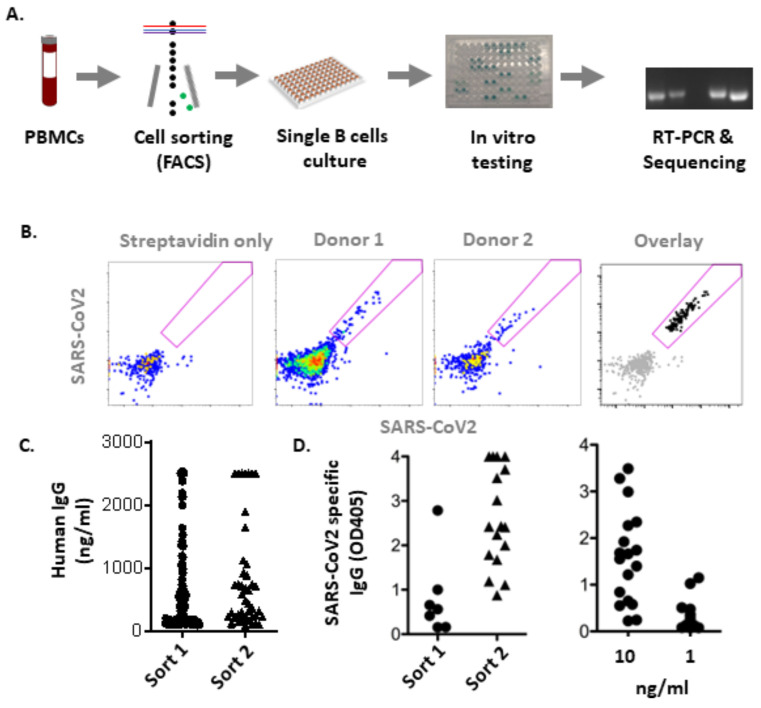
Isolation of human mAbs against SARS-CoV-2 S protein. (**A**) Workflow of isolation procedure and (**B**) representative FACS plots for specific B cell sort are depicted. (Left) sorted B cells (black) were overlaid onto unstained B cells. (**C**) Total IgG levels, independent of antigen specificity, from supernatants of sorted single B cell culture were analyzed by ELISA. (**D**) Binding affinity against the SARS-CoV-2 S protein of mAbs from single B cell culture was analyzed at 50 ng/mL (left) as well as 10 and 1 ng/mL (right).

Producing large numbers of mAb candidates in sufficient quantity and quality for preclinical studies is challenging. Here, we sought to facilitate the in vivo screening of both individual mAb candidates and mAb cocktails using naked DNA-encoded modified antibodies. We selected four human mAbs with distinct binding characteristics, Ab100, MPE8, 15C3 and 37C4. Ab100 and MPE8 were previously shown to reduce lung viral titers of both RSV and HMPV in relevant mouse models [13,34]. Using fluorescent activated cell sorting (FACS), we isolated 15C3 and 37C4 from the PBMCs of two convalescent individuals following SARS-CoV-2 infection (Figure 1). These convalescent individuals were sampled in the first trimester of 2020, prior to the emergence of SARS-CoV-2 variants of concern [35].

**Figure 2:**
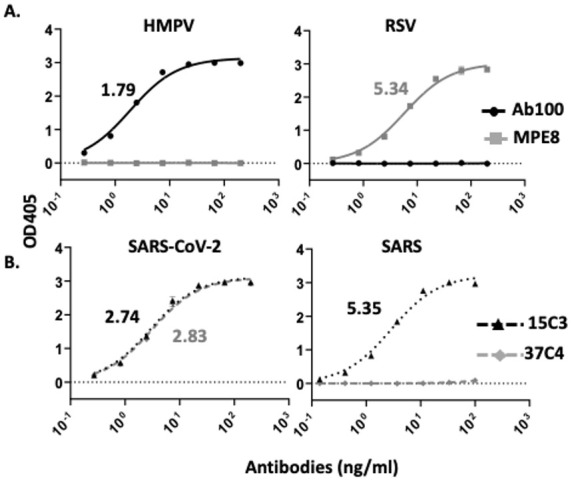
Binding EC_50_ of developed scFV-IgG. The binding EC_50_ of scFV-IgG versions of Ab100, MPE8 (**A**), 15C3 and 37C4 was measured by ELISA (**B**) against the fusion (F) proteins or RSV and HMPV (**A**) or the spike (S) protein of SARS-CoV-2 or SARS (**B**). Binding EC_50_ values in ng/mL are indicated. Each sample was run in triplicate. Means +/− standard deviation (SD) are depicted.

The administration of a cocktail of conventional DMAb would generate non-functional mAbs with mismatched heavy and light chains. To prevent this, the four above-mentioned mAbs were modified into scFv-IgG. Codon-optimized constructs, including the variable domain of the light chain (V_L_) of each mAb, a glycine serine linker (G_4_S), the variable domain of its corresponding heavy chain (V_H_) and the Fc region of a human IgG1, were synthesized [29,30]. For each mAb, the resulting scFv-IgG construct was cloned into our custom plasmid vector (pIDV-II) [31]. Next, HEK 293 cells were individually transfected with each of four generated DNA-scFv-IgG. The amount of human scFv-IgG produced in the supernatant was quantified by ELISA. The binding characteristics of the generated scFv-IgG were then monitored by ELISA. The Ab100 scFv-IgG strongly bound to the HMPV fusion (F) protein (1.79 ng/mL EC50) but did not recognize the monomeric RSV F protein. Conversely, the MPE8 scFv-IgG did not bind to the HMPV F protein but interacted with high affinity with the RSV F protein (5.34 ng/mL EC50) (Figure 2A). Both 15C3 and 37C4 scFv-IgG targeted the spike (S) protein of SARS-CoV-2 with a strong binding EC50 of 2.74 and 2.83 ng/mL, respectively. However, only the 15C3 scFv-IgG bound to SARS S protein (5.35 ng/mL EC50) (Figure 2B). Of note, despite both 15C3 and 37C4 targeting the receptor binding domain (RBD) of the S protein, neither scFv-IgG neutralizes SARS-CoV-2 (Figures S1 and S2). While unexpected, the lack of neutralization by both scFv-IgGs might be due to their binding epitopes not fully overlapping with SARS-CoV-2 RBD or these epitopes not being fully accessible on the surface of SARS-CoV-2 virions. Indeed, viral surface proteins undergo conformation changes that can expose novel epitopes or bury existing ones [36]. Of note, both scFv-IgG possess a full Fc region. In vivo, these scFv-IgGs are expected to decrease SARS-CoV-2 viremia via Fc effector functions, such as antibody-dependent cellular cytotoxicity (ADCC) and complement-dependent cytotoxicity (CDC) [36,37].

### 3.2. Multiple DNA-scFv-IgG Can Be Administered In Vitro and In Vivo

**Figure 3:**
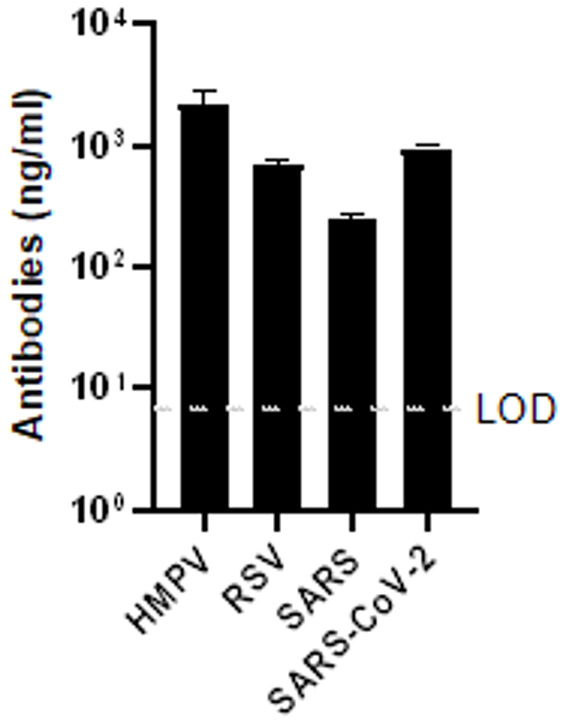
Secreted scFV-IgG level following co-transfection. HEK293 cells in 12-well plates were transfected with 250 ng each of DNA-scFV-IgG encoding Ab100, MPE8, 15C3 and 37C4 scFv-IgG. Secreted levels of scFv-IgG against each of the target antigens were measured by ELISA. The assay limit of detection (LOD) is indicated by a dotted line. Mean +/− SD of individual samples ran in triplicate are depicted.

Next, the ability to co-administer the four DNA-scFv-IgGs was investigated, both in vitro and in naïve mice. First, HEK293 cells in 12-well plates were co-transfected with 250 ng each of the four DNA-scFv-IgGs. Non-transfected cells were used as negative controls. The amount of each scFv-IgG in the supernatant, 4 days post-transfection, was monitored by ELISA using standard curves made of individual scFv-IgG. On average, 2.13, 0.69, 0.25 and 0.95 µg/mL of antibodies against HMPV, RSV, SARS-1 and SARS-CoV-2 were detected (Figure 3). While the produced amount of each scFv-IgG greatly varies, each of the four scFv-IgGs could be readily detected following co-transfection.

**Figure 4:**
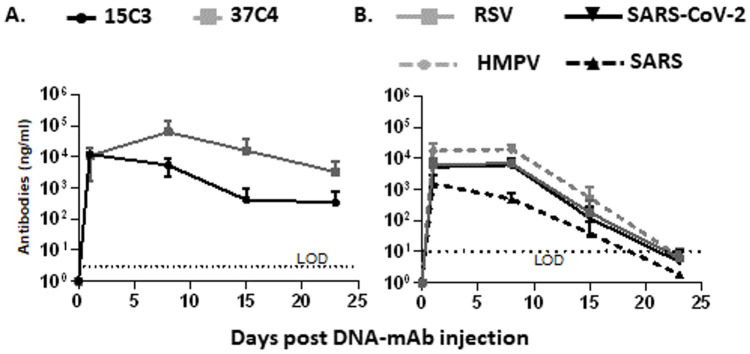
Serum level of scFv-IgG following hydrodynamic administration of DNA-mAb. 10 µg of individual DNA-scFv-IgG (**A**) or a 20 µg cocktail of 4 DNA-scFv-IgG (5 µg each) (**B**) were administered hydrodynamically via the retroorbital route to female BALB/c mice (n = 3/group). Serum levels of the generated scFv-IgG were monitored before and after injection (day 1, 8, 15 and 23). LOD: assay limit of detection. The mean +/− SD of each sample ran in triplicate is depicted.

Next, the ability to administer individual DNA-scFv-IgG or a cocktail of the four DNA-scFv-IgGs was evaluated in naïve mice. Female BALB/c mice each received 10 µg of DNA-scFv-IgG encoding 15C3 or 37C4. Naked DNA-scFv-IgGs were delivered hydrodynamically via the retroorbital route, as previously reported [25]. Mice were bled 7 days before and 1, 8, 15, and 23 days after DNA-scFv-IgG administration. Serum levels of the different scFv-IgGs were measured by ELISA using standard curves made of individual scFv-IgG, as described above. On average, 12.1 +/− 1.5 and 10.9 +/− 9.2 µg/mL (mean +/− SD) of 15C3 and 37C4 scFv-IgG were detected 24 h post-DNA-scFv-IgG injection. Levels of 15C3 scFv-IgG steadily declined to 5.5, 0.4 and 0.3 µg/mL on days 8, 15 and 23 post-DNA-scFv-IgG administration. In contrast, levels of 37C4 increased to 62.9 µg/mL on day 8 and decreased to 16.0 and 3.3 µg/mL on days 15 and 22, respectively (Figure 4A). In a previous study, following the hydrodynamic delivery of plasmid DNA, antibody levels increased for approximately 24 h then remained stable until the last evaluated timepoint, 4 days post-delivery [38]. Higher serum levels of 37C4 scFv-IgG on day 8 compared to day 1 might indicate that 37C4 scFv-IgG serum levels were still rising after the day-1 timepoint and later reached a plateau, rather than steadily increasing from day 1 to day 8.

The impact of DNA-scFv-IgG co-administration on circulating scFc-IgG levels was then monitored. Following hydrodynamic delivery, no increase in transgene expression was detected above 5 ug of plasmid in previous studies [24,25]. So, individual mice were injected hydrodynamically via the retroorbital route with a cocktail of 5 µg, the maximum dose, of each of DNA-scFv-IgG encoding 15C3, 37C4, Ab100 and MPE8 (20 µg total). Once more, mice were bled prior to DNA-scFv-IgG injection, then weekly starting on day 1 after injection, and scFv-IgG levels were determined by ELISA. Peak circulating levels of the different scFv-IgGs were observed on day 1 or 8 post-DNA-scFv-IgG injection, with peak levels of scFv-IgG against HMPV, RSV, SARS-1 and SARS-CoV-2 at 19.5, 6.7, 1.5 and 6.0 µg/mL, respectively. Of note, there was a sharp decline in the circulating levels of all scFv-IgG 15 and especially 22 days post-DNA-scFv-IgG injection (Figure 4B).

Overall, serum levels of scFv-IgG above 1.5 µg/mL were detected following naked DNA-scFv-IgG administration, suggesting that the HD of the DNA-mAb cocktail was suitable for challenge studies in mice.

### 3.3. A DNA-scFv-IgG Cocktail Can Protect Mice in Various Models of Infection

**Figure 5:**
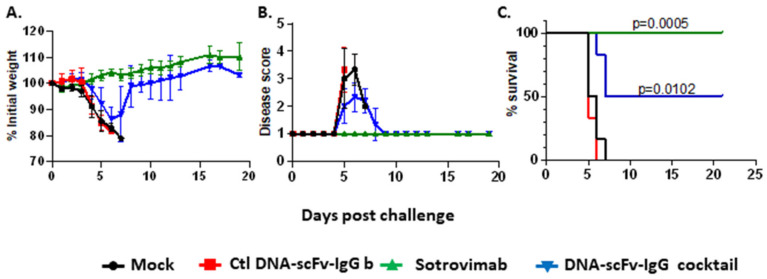
A 4 DNA-mAb cocktail is partially protective in the SARS-CoV-2 model of infection. K18-hACE2 mice were treated hydrodynamically with 20 µg of control DNA-scFv-IgG (day-3) (red lines) or a cocktail of 4 DNA-scFv-IgG (5 µg each) (day-3) (blue lines), or intraperitoneally with 200 µg of sotrovimab (day-1) (green lines). Naive mice were used as control (black lines). All mice (n = 6/groups) were challenged intranasally with 6 × 10^4^ TCID50 of SARS-CoV-2 (USA-WA1/2020). Animals were monitored for weight loss (**A**), signs of diseases (**B**) and survival (**C**). *p* values for statistically significant differences between the control DNA-scFv-IgG group and the treated groups are indicated. (**A**,**B**) Mean +/− SD are illustrated.

Finally, we assessed whether the HD of the four developed DNA-scFv-IgGs could protect mice against SARS-CoV-2 and RSV challenge. The human ACE2 transgenic mice (K18-hACE2) model of SARS-CoV-2 infection was used [39]. K18-hACE2 mice were either treated with 20 µg of a control DNA-scFv-IgG or 20 µg of the above-described DNA-scFv-IgG cocktail (5 µg of each DNA-scFv-IgG) three days before challenge. Groups of naïve mice and mice treated, 1 day before challenge, with 200 µg (10 mg/kg) of Sotrovimab were used as negative and positive controls, respectively [40]. All mice were challenged intranasally with 6 × 10^4^ TCID50 of SARS-CoV-2 (USA-WA1/2020). The weight loss and signs of disease were monitored for 21 days post-viral challenge. All control mice, either naïve or treated with control DNA-scFv-IgG, were euthanized between days 5 and 7 post-challenge due to weight loss above 20% or reaching the humane end point (Figure 5A–C). In contrast, none of the Sotrovimab-treated mice lost more than 5% of their initial weight or showed signs of disease. Four of the six animals treated with the DNA-scFv-IgG cocktail showed signs of disease, with three of them reaching the end point for euthanasia, resulting in a 50% survival rate (Figure 5A–C). In the DNA-scFv-IgG cocktail group, survival might correlate with the level of scFv-IgG following hydrodynamic delivery. However, none of the SARS-CoV-2-challenged animals were sampled during the course of the experiment, as this would have affected animal survival. In infected animals, the additional stress from anesthesia and extra handling could have negatively impacted survival. As mentioned above, previous studies did not observe any increase in transgene expression following hydrodynamic delivery of more than 5 ug of plasmid [24,25]. As a result, we do not anticipate that increasing the amount of DNA-scFv-IgG would improve protection efficacy.

**Figure 6:**
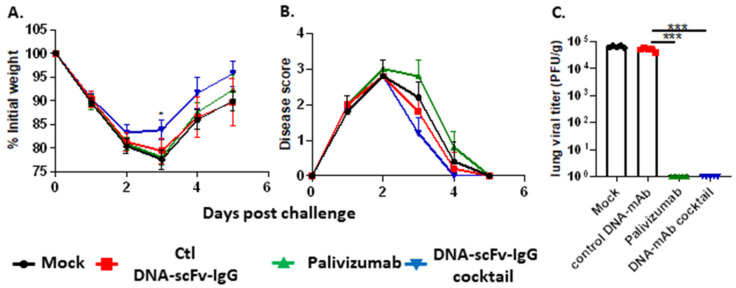
In vivo inhibition of RSV replication by a 4 DNA-mAb cocktail. Female BALB/c mice (n = 5/group) were mock treated (black circle), treated with 20 µg of control DNA-scFv-IgG (day-3) (red square), 200 µg of palivizumab (day-1) (green triangle) or 20 µg of our DNA-scFv-IgG cocktail (day-3) (blue triangle). Animals were challenged with 5 × 10^6^ PFU of RSV and monitored for weight loss (**A**), clinical signs of disease (**B**) and lung viral titers 5 days post-challenge (**C**). Statistically significant differences between the control DNA-mAb group and the treatment groups are depicted with *p* values below 0.05 and 0.0001 indicated by * and ***, respectively. (**A**–**C**) Mean +/− SD are depicted.

The protective efficacy of the same DNA-scFv-IgG cocktail was also tested in RSV-challenged BALB/c mice. Mock and irrelevant DNA-scFv-IgG-treated mice were used as negative control, with mice treated with 100 µg (5 mg/kg) of Palivizumab, 24 h before challenge, used as positive control. Both irrelevant DNA-scFv-IgG and the DNA-scFv-IgG cocktail (20 µg/mice) were injected hydrodynamically via the retroorbital route 3 days before challenge. All animals were challenged with 5 × 10^6^ PFU of RSV (strain pool G493) and monitored for weight loss and signs of disease for 5 days prior to euthanasia. All RSV-infected animals showed signs of disease and weight loss (Figure 6A,B). Lung viral titers above 5 × 10^4^ PFU/g were observed in naïve mice and mock-treated mice. In contrast, no virus was detected in mice that received Palivizumab or the DNA-scFv-IgG cocktail (Figure 6C). Of note, lung viral titers were measured 5 days post-RSV infections. In the Palivizumab and DNA-scFv-IgG cocktail-treated mice, the lack of detectable lung viral titers 5 days post-RSV infection does not suggest a lack of viral replication at earlier timepoints. Instead, in these groups of mice, viral clearance occurs more quickly than in mock and control DNA-scFv-IgG-treated animals, as indicated by the weight loss in all RSV-challenged mice. Overall, the above data confirm that the HD of the DNA-scFv-IgG cocktail induces high serum levels of the individual scFv-IgG and that these systemic scFv-IgG levels can be protective in mouse models of infections.

## 4. Discussion

Screening mAb candidates in animal models is necessary to inform us about their therapeutic efficacy. This study utilized DNA-scFV-IgGs administered hydrodynamically via the retroorbital route to streamline the in vivo screening of therapeutic mAbs. HD requires 10 µg or less of individual DNA-scFv-IgG, making a simple maxiprep sufficient to test the efficacy of each mAb. In contrast, producing mAbs, in house or through specialized companies, is costly and time consuming. The described technique significantly reduces costs and accelerates the in vivo screening process for therapeutic mAbs. While this study focuses on rapid in vivo screening, the in vitro identification of lead candidates is still required prior to animal experiments. The mAb cocktail used in this study is of limited therapeutic value. The mAb selection was primarily driven by the distinct and well-defined binding properties, allowing the expression of the four distinct scFv-IgGs to be readily measured.

In the present study, HD was performed prophylactically. The rapid rise of circulating scFv-IgG following DNA-scFv-IgG HD suggests that this method could also be used for therapeutic evaluation. In that regard, DNA-scFv-IgG HD is similar to the administration of mAb (protein) that increases the mAb serum concentration within hours, followed by a gradual decline [41,42]. In contrast, the intramuscular delivery of DMAbs followed by electroporation slowly increases mAb circulating levels, with the peak concentration 2–3 weeks post-administration [43,44,45].

Using HD, repeat plasmid DNA administration was reported [24,46]. Thus, repeated DNA-scFV-IgG dosing should be possible. As a proof of concept, a cocktail of mAbs targeting different pathogens was tested. Cocktails of DNA-scFV-IgG targeting the same viral protein or distinct proteins of the same pathogens can also be screened. It is worth mentioning that HD via the mouse tail vein was previously used to study the impact of isotype switching on the therapeutic efficacy of an influenza virus-specific mAb [38]. However, retroorbital injections are less technically challenging than tail vein injections [25,47]. Indeed, our group was unable to consistently perform tail vain injections, highlighting the technical challenges associated with tail vain injections. Transforming mAb into scFv-IgG can sometimes significantly reduce the binding affinity [48]. In those instances, scFv-IgG with low binding affinity would be excluded from further downstream screening. Furthermore, in mice, the serum level of different scFv-IgGs can vary by more than 1 log. Differences in scFv-IgG expression will influence the therapeutic efficacy of the tested mAb candidates. It is worth pointing out that, in mice, low systemic antibody levels following DNA-scFv-IgG delivery via HD are likely indicative of mAbs with a low yield. In this study, the in vitro and in vivo production yield of the four tested scFv-IgGs correlated. As the production yield is a crucial criterion in the developability assessment of therapeutic mAb candidates, the early exclusion of mAbs with a low expression yield would be beneficial [49,50]. Once protective mAbs have been identified, either the scFv-IgG or IgG version of these mAbs could be used for further clinical development.

HD via the tail vein or the retroorbital route triggers DNA accumulation in the liver and subsequent transgene expression in treated animals [24,25,51]. A similar method, denoted hydrodynamic limb vein (HLV) injection, was also described for transgene expression in distal muscles [46,52]. Using the HLV delivery of naked plasmid DNA, robust transgene expression was demonstrated in the limb muscles of rats, dogs, and rhesus macaques [46,52]. This suggests that combining DNA-scFV-IgG and HLV delivery could be used to screen the therapeutic efficacy of therapeutic mAbs in larger mammal species, including non-human primates (NHPs). Of note, the combination of DNA-scFV-IgG and HD is applicable to the screening of mAb candidates against not only infectious diseases but also cancers and autoimmune diseases.

In summary, the current study demonstrates that DNA-scFv-IgGs administered hydrodynamically could be a valuable tool to speed up and decrease the cost of the in vivo screening of therapeutic mAb candidates. Using this format, the therapeutic efficacy of both individual mAbs and mAb cocktails could be rapidly validated in animal models of infections, limiting cumbersome mAb manufacturing to the lead candidates. Our study warrants further optimization of DNA-scFv-IgG delivery in humans for the clinical application of antibody gene therapy.

## Data Availability

Data is contained within the article or Supplementary Materials.

## Supplementary Materials

The following supporting information can be downloaded at: https://www.mdpi.com/article/10.3390/biomedicines13030637/s1, Figure S1: 15C3 and 37C4 binding to the receptor binding domain of SARS-CoV-2 spike protein. Figure S2: Neutralization capacity of 15C3 and 37C4 against SARS-CoV-2.
