# Supplementary material for: Rapid In Vivo Screening of Monoclonal Antibody Cocktails Using Hydrodynamic Delivery of DNA-Encoded Modified Antibodies
Source: Biomedicines. 2025 Mar 5;13(3):637. doi: 10.3390/biomedicines13030637 (PMC11940352; doi:10.3390/biomedicines13030637)
Supplement: Supplementary file 1 [file biomedicines-13-00637-s001.zip › biomedicines-3488704-supplementary.pdf]

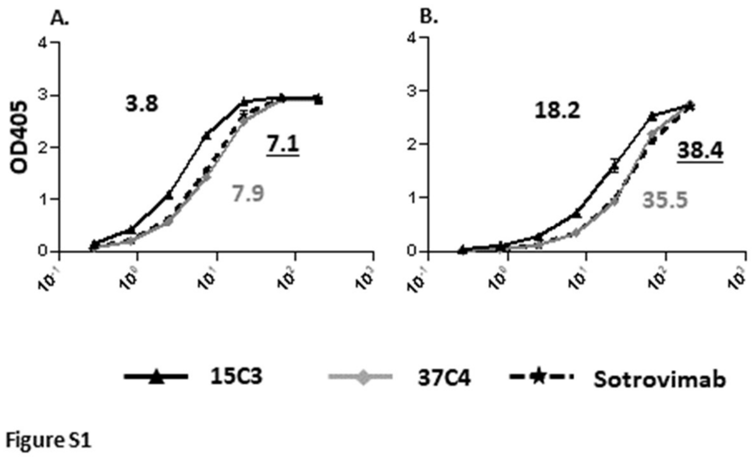

Figure S1: 15C3 and 37C4 binding to the receptor binding domain of SARS-CoV-2 spike protein Binding ELISA of mAb transfection supernatants (15C3, 37C4) or purified mAb (Sotrovimab) were assessed for binding to the receptor binding domain (RBD) of SARS-CoV-2 Wuhan (**A**.) or Beta variant (**B**.). Binding EC_{50} in ng/ml of 15C3 (black), 37C4 (grey) and Sotrovimab (underlined) are indicated. Each sample was run in triplicate. Mean +/- SD are depicted.

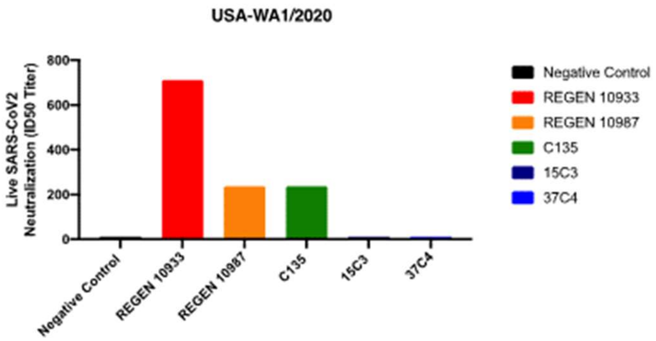

Figure S2: Neutralization capacity of 15C3 and 37C4 against SARS-CoV-2

The neutralization ID50 titers were determined for 15C3 and 37C4 scFV-IgG, in comparison with REGN 10933, REGN 10987, and C135 mAb controls. Serial dilutions of each mAb were incubated with 300 TCID50/mL of virus and incubated for 5 days. Wells were scored as positive or negative for CPE and the neutralization ID50 titer was calculated.
